# Is the Macrophage Phenotype Determinant for Fibrosis Development?

**DOI:** 10.3390/biomedicines9121747

**Published:** 2021-11-23

**Authors:** Lluis Lis-López, Cristina Bauset, Marta Seco-Cervera, Jesús Cosín-Roger

**Affiliations:** 1 Department of Pharmacology and CIBEREHD, Faculty of Medicine, University of Valencia, 46010 Valencia, Spain; lluislis@alumni.uv.es (L.L.-L.); cristina.bauset@uv.es (C.B.); 2 Hospital Dr. Peset, Fundación para la Investigación Sanitaria y Biomédica de la Comunitat Valenciana, FISABIO, 46010 Valencia, Spain; jesus.cosin@uv.es

**Keywords:** macrophages, pulmonary fibrosis, cardiac fibrosis, liver fibrosis, kidney fibrosis, intestinal fibrosis

## Abstract

Fibrosis is a pathophysiological process of wound repair that leads to the deposit of connective tissue in the extracellular matrix. This complication is mainly associated with different pathologies affecting several organs such as lung, liver, heart, kidney, and intestine. In this fibrotic process, macrophages play an important role since they can modulate fibrosis due to their high plasticity, being able to adopt different phenotypes depending on the microenvironment in which they are found. In this review, we will try to discuss whether the macrophage phenotype exerts a pivotal role in the fibrosis development in the most important fibrotic scenarios.

## 1. Introduction

Against an injury, tissues physiologically respond with a complex process called wound healing in order to remove the detrimental stimuli. Nevertheless, if the damage persists and becomes chronic, a non-physiological process named fibrosis comes into play [1]. Tissue fibrosis is characterized by an excessive formation and deposition of extracellular matrix (ECM), leading to the alteration of the architecture and function of the organ. Basically, fibrosis comprises the same mechanisms involved in the physiological wound healing response but becomes intensified given the chronic persistence of the harmful stimuli. Hence, fibrogenic responses cause a shift from a profitable wound healing in order to resolve the injury, towards an excessive ECM deposition resulting in an extensive scar formation [2]. Indeed, it has been reported that in such conditions, collagen microarchitecture appears thickened and distorted, leading to an abnormal ECM structure that has effects on the surrounding cell population, including myofibroblasts [3].

This pathological process can be developed in several organs such as liver, lung, kidney, heart, and intestine, and it plays a central role in the progression of many chronic diseases. In fact, this scarring process leads to mortality of approximately 45% of the population in the developed world [4].

Fibrosis is initiated by the presence of injurious agents that cause the destruction of parenchymal cells through necrosis, apoptosis, pyroptosis, necroptosis, and ferroptosis [5,6,7,8,9]. This tissular damage is accompanied by the activation of the inflammatory response and the arrival of several types of blood cells to the injury. Next, both local and new immune cells secrete a wide variety of cytokines and chemokines, triggering the activation of the mesenchymal cells, which produce ECM and further enhance the release of pro-inflammatory cytokines, angiogenic factors, and chemokines. The activation of these mesenchymal cells from a quiescent towards an active status is characterized by the expression of α-smooth muscle actin (α-SMA) [10]. In addition, the number of cells producing ECM is significantly increased given the ability of several cells that can switch their phenotype and become active myofibroblasts. Of interest, recent studies that used novel computational approaches have reported that myofibroblast differentiation and fibrosis can also be influenced by alterations in ECM microarchitecture [3]. Indeed, besides resident fibroblasts, mesothelial cells, pericytes, and circulating fibrocytes, even epithelial cells, endothelial cells, and macrophages can acquire a myofibroblast phenotype through epithelial–to–mesenchymal transition (EMT), endothelial–mesenchymal transition (EndoMT) and macrophage–myofibroblast transition (MMT), respectively [11,12].

The key protagonist of the fibrosis in all organs is the transforming growth factor-beta (TGF-β). So far, three different isoforms of TGF-β have been identified: TGF-β1, TGF-β2, and TGF-β3, although TGF-β1 is the main participant in physiological repair, collagen accumulation, and fibrosis induction [13]. This cytokine, in order to be recognized by its receptor, TGF-β receptor, which is a heterotetrameric complex, needs to be cleaved since it is secreted as an inactive molecule. Once TGF-β binds to the receptor, it activates both canonical and non-canonical signaling pathways [14]. On the one hand, in the canonical pathway, the receptor recruits and induces the phosphorylation of the proteins SMAD2 and SMAD3, which in turn associate with the protein SMAD4, forming a transcriptional factor that enters into the nucleus and activates the expression of several genes. On the other hand, in the non-canonical pathway, the receptor recruits different adaptor proteins such as growth factor receptor-bound protein 2 (Grb2), Src homology and collagen A protein (ShcA), or tumor necrosis factor receptor-associated factor 6 (TRAF6), which leads to the phosphorylation of several kinases such as the extracellular signal-regulated kinase (Erk), protein kinase B (Akt), c-Jun N-terminal kinase (JNK), and p38 [15,16]. In spite of the fact that TGF-β is considered the master regulator of fibrosis, it also plays essential roles in numerous biological processes such as angiogenesis, cell differentiation, immune tolerance, etc. Therefore, it is important to take into account this pleiotropic role of the cytokine since its targeting might cause a wide range of side effects. This is the reason why emerging pharmacological compounds are designed against other molecules involved in fibrosis development.

As we have previously mentioned, there is a wide range of different cell types involved in the complex process of fibrosis. Although at first glance it seems that fibroblasts are the main protagonists of this pathological process, it is important to highlight that immune cells are also essential in fibrosis induction. In fact, among immune cells, macrophages play a key role not only in the first activation of the inflammatory pathways against the harmful stimuli, but also in the regeneration and activation of numerous cells through all the molecules secreted by them. Remarkably, recent studies have demonstrated that disturbances in ECM composition, including stiffness, bulking, and the presence of biopolymers from collagen I, can even affect macrophages’ polarization, and thus, their role in fibrosis modulation [17]. Therefore, given the importance of these cells in fibrosis induction, in the present review we will describe the role of macrophages in the most common fibrotic scenarios and, given their plasticity, we will also emphasize whether the specific macrophage phenotype is determinant in the activation of the fibrotic pathways.

## 2. Macrophage Polarization

Macrophages are versatile cells that exhibit a high degree of plasticity. The process through which macrophages obtain distinctive functional features as a response to certain stimuli from their microenvironment is known as macrophage polarization [18]. When tissue is injured, macrophages are pushed towards a pro-inflammatory phenotype that should be followed by the switching of these cells to a wound healing phenotype that promotes ECM production by myofibroblasts, and eventually, polarizing to a pro-remodeling phenotype that is required to ensure restoration of physiological tissue composition [19]. Recently, single-cell RNA-sequencing has allowed the identification of two different origins for macrophages that populate tissues, i.e., tissue-resident macrophages derived from embryonic progenitors, and monocyte-derived macrophages (MoMFs) [20,21]. The first ones arrive on site during organ development and can be self-maintained, whereas the second ones migrate from blood to a tissue upon injury and are able to differentiate into macrophages [22]. Recently, it has been described that these MoMFs can assume a phenotype similar to tissue-resident macrophages instead of dying, which reinforces the tissue microenvironment role in the macrophage regulatory landscape [23]. A relevant implication of this polarization by MoMFs in fibrotic phenomena has been demonstrated in a large number of studies; specifically, several fibrotic mouse models have shown fibrosis attenuation after their depletion [24,25,26].

**Figure 1:**
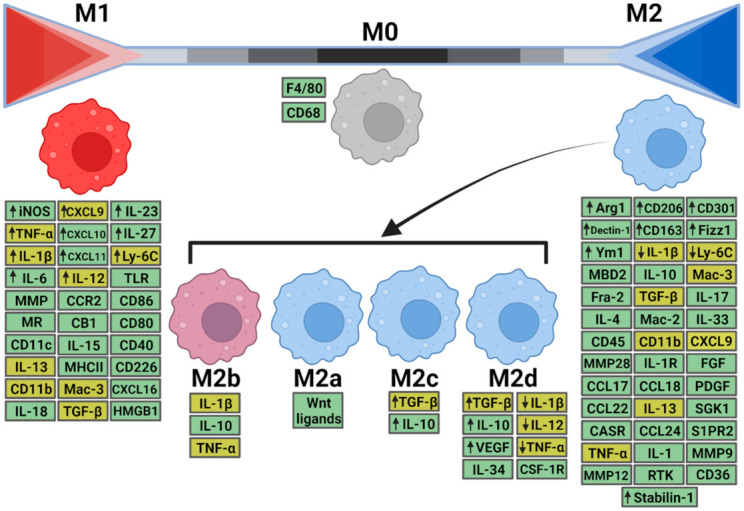
Presence of the main markers found in the different macrophage subtypes. Markers in light green are found in both types of macrophages (M1 and M2). Arrows indicate increased (↑) or decreased (↓) levels of the marker. Macrophage pro-inflammatory (M1), Macrophage anti-inflammatory/pro-fibrotic (M2), Inducible nitric oxide synthase (iNOS), Chemokine C-X-C motif ligand (CXCL), Interleukin (IL), Tumor necrosis factor-alpha (TNF-α), Lymphocyte antigen 6 complex, locus C1 (Ly6C), Toll-like receptor (TLR), Matrix metalloproteinase (MMP), CD (Cluster of differentiation), Mineralocorticoid receptor (MR), Cannabinoid receptor 1 (CB1), Class II major histocompatibility complex (MHCII), Transforming Growth Factor-beta (TGF-β), High-mobility group box-1 (HMGB1), Arginase 1 (Arg1), Mannose receptor 1 (CD206), Resistin-like protein α (Fizz1), Methyl-CpG-binding domain 2 (MBD2), Fos-related antigen-2 (Fra-2), Fibroblast growth factor (FGF), Chemokine C-C motif ligand (CCL), Platelet derived growth factor (PDGF), Serum/Glucocorticoid Regulated Kinase 1 (SGK1), Class A scavenger receptor (CASR), Sphingosine-1-phosphate receptor-2 (S1PR2), Receptor tyrosine kinases (RTK), Vascular endothelial growth factor (VEGF), Colony-stimulating factor-1 receptor (CSF-1R).

Stimulated by appropriate factors or the tissue microenvironment, macrophage polarization leads to generation of distinct subsets of macrophages, namely classically activated M1 (pro-inflammatory) and alternatively activated M2 (anti-inflammatory/pro-fibrotic) macrophages. However, it is known that this classification is more complex, and macrophage behaviors are better described as a series of gradations within a large spectrum [27]. Therefore, M2 macrophages can be sub-classified into M2a, M2b, M2c, and M2d based on the secretion of distinct cytokines, presence of certain cell surface proteins, gene expression profiles and other biological activities (Figure 1) [28].

A growing body of data highlights the importance of metabolism in the regulation of macrophage polarization. For instance, arginine metabolism plays an important role in this process since two opposed pathways, inducible nitric oxide synthase (iNOS) pathway and arginase pathway, are involved in M1 and M2 polarization, respectively. Activation of the iNOS pathway generates citrulline and nitric oxide (NO) from arginine, promoting M1 macrophage differentiation, whereas activation of the arginase pathway enhances the production of ornithine and urea from arginine, increasing the number of differentiated M2 macrophages [29]. Similarly, glucose availability and metabolic conversion to pyruvate and lipogenesis are important for the polarization of M1 macrophages, whereas the roles of glycolysis and fatty acid oxidation in the differentiation of M2 macrophages are still controversial [30]. Alterations of the tricarboxylic acid (TCA) cycle are related to M1 macrophages and are a consequence of two reactions, catalyzed by isocitrate dehydrogenase and succinate dehydrogenase, that lead to the accumulation of citrate and succinate [31]. Excessively produced citrate causes an increase in the generation of pro-inflammatory molecules such as NO and prostaglandin [32]. Succinate is another metabolite accumulated from the broken TCA cycle and associated with the pro-inflammatory function of M1 macrophages [33,34]. By contrast, glutamine formation from glutamate is important to M2 macrophage function, pointing out the relevance of glutamine metabolism in this polarization [35].

In addition to the metabolic pathways involved in macrophage polarization, different molecules are described as activators or markers of M1 or M2 polarization (Figure 1).

### 2.1. Profiles of M1 Macrophages

M1 polarization is classically induced by lipopolysaccharide (LPS) and Th1 cytokines such as interferon gamma (IFN-γ) and tumor necrosis factor alpha (TNF-α). Additionally, granulocyte-macrophage colony-stimulating factor (GM-CSF) has been described as an important M1 inducer [36]. This subset of macrophages is characterized by the expression of surface markers CD40, CD80, and CD86, which promotes cytotoxic adaptive immunity, in conjunction with class II major histocompatibility complex (MHCII), and toll-like receptors (TLR) 2 and 4. Moreover, these macrophages synthesize increased levels of iNOS and a number of cytokines and chemokines: inflammatory cytokines such as TNF-α, interleukin (IL)-1β, IL-6, Th1, and Th17 orientating cytokines such as IL-12, IL-27, and IL-23, and Th1-recruiting chemokines such as chemokine (C-X-C motif) ligand (CXCL) 9, CXCL10, and CXCL11 that induce further polarization of M1 macrophages via positive feedback (more deeply reviewed in [37]).

The main transcription factors activated in M1 polarization and responsible for their characteristic gene expression profile are nuclear factor-kappa B (NF-κB), signal transducer and activator of transcription 1 (STAT1), interferon regulatory factor (IRF) 3 and 5, hypoxia induced factor 1 alpha (HIF1α), and activator protein 1 (AP1). NF-κB is activated by TLRs, interleukin 1 receptor (IL-1R), and TNF-α, promoting the expression of pro-inflammatory pathways [38,39]. Similarly, IFN-γ via the JAK/STAT pathway induces STAT1 activation, which results in an elevated expression of pro-inflammatory cytokines and, therefore, leads macrophages to M1 polarization [40]. Stimulation of TLR4 or TLR3 also activates IRF3 and induces transcription of the IFN-β gene to form the type I IFN loop for optimal M1 activation of macrophages [41]. IRF5, activated by pro-inflammatory factors, enhances the IFN-γ/JAK/STAT1-dependent production of IL-12 [42]. In addition, IFN-γ promotes HIF1α accumulation, which enhances the expression of several genes including those for inflammatory cytokines such as TNF-α, IL-1β, IL-6, IL-12, effectors against bacteria such as iNOS, glycolytic enzymes such as phosphoglycerate kinase, and glucose transporters such as glucose transporter 1 (Glut1) [43]. AP1 is a heterodimer composed of proteins including c-Fos and c-Jun families. Activation of JNK by TNF-α produces the phosphorylation of c-Jun and consequently heterodimerization of c-Jun/c-Fos, which finally leads to the activation of pro-inflammatory genes [44].

### 2.2. Profiles of M2 Macrophages

Induction of M2 polarization is directly exerted by the anti-inflammatory cytokines IL-4 and IL-13 and macrophage colony-stimulating factor (M-CSF). However, other cytokines such as IL-10, IL-33, and TGF-β can also induce polarization of macrophages towards this phenotype [45]. In contrast to the M1 characteristic gene expression phenotype, M2 macrophages present an anti-inflammatory profile characterized by the presence of anti-inflammatory molecules such as IL-10, TGF-β, and IL-1R type 1 and 2. Moreover, these macrophages present high expression levels of receptors CD206, CD301, dectin-1, CD163, Stabilin-1, resistin-like protein α (FIZZ1), and YM1 (more deeply reviewed in [30]). In addition, the recruitment of Th2 and Treg cells, eosinophils, and basophils is mediated by the secretion of chemokines chemokine (C-C motif) ligand CCL17, CCL18, CCL22, and CCL24 by M2 macrophages [46]. When M2 polarization is induced by IL-4 and IL-13, the phenotype of these macrophages is known as M2a, whereas other M2 behaviors can be induced by different stimuli. Indeed, M2b, or regulatory macrophages, are induced by immune complexes and TLR ligands or by IL-1R agonist. This phenotype produces pro- and anti-inflammatory cytokines, such as IL-1β, IL-10, and TNF-α, hence regulating immune and inflammatory response. The third subtype of macrophages with a strong anti-inflammatory profile is known as M2c. These are activated by glucocorticoids or IL-10, and present increased secretion levels of IL-10 and TGF-β. Regarding this, increased secretion of IL-10 by macrophages, specifically M2c, was associated with good resolution of wound healing in in vitro and in vivo studies [47,48,49]. The last M2 phenotype, named M2d, is associated with tumor angiogenesis, growth, and metastasis. M2d macrophages or tumor-associated macrophages (TAM) secrete elevated levels of IL-10, TGF-β, and vascular endothelial growth factor (VEGF), and decreased levels of IL-12, TNF-α, and IL-1β [50].

M2 polarization is characterized by the expression of transcription factors that regulate the specific expression profile of this phenotype. Among them, STAT6, IRF4, Jumonji domain containing-3 (JMJD3), and peroxisome proliferator-activated receptor (PPAR)-δ and -γ are the main transcription factors. IL-4 and IL-13 binding could regulate tyrosine phosphorylation on the IL-4 receptor-α (IL-4Rα) cytoplasmic tail to accelerate recruitment and further tyrosine phosphorylation of STAT6 by JAK1/JAK3 or JAK1/Tyk2, respectively [51]. Consequently, the STAT6 homodimerization is enhanced and results in the recruitment of IRF4 and the activation of target genes associated with the M2 phenotype [52]. Furthermore, a histone 3 lysine-27 demethylase (H3K27), JMJD3, was found to regulate expression of the transcription factor IRF4, promoting M2 polarization [53]. PPARδ and PPARγ are induced by IL-4 and IL-13 ligands through the STAT6 pathway, regulating the M2 phenotype [54]. Additionally, PPARγ produces inhibitory effects on the pro-inflammation profile through a post-transcriptional sumoylation that protects ubiquitin-mediated proteasomal degradation of the nuclear receptor corepressor–histone deacetylase-3 complex. This stabilization maintains the promoter-specific repressor of NF-κB target genes that regulates immunity and homeostasis [55].

## 3. Macrophages in Lung Fibrosis

Pulmonary fibrosis is a lung disease that includes a wide variety of heterogeneous disorders. This occurs when lung tissue is damaged and causes scars that ultimately lead to organ malfunction [56]. Pulmonary fibrosis is associated with many pulmonary pathologies. In fact, idiopathic pulmonary fibrosis (IPF) is the most common [57], with a 5-year survival rate of 20% to 30% [58]. This group of pathologies is characterized by inflammation, scarring, thickening, and stiffness of the alveolar walls. Nevertheless, pulmonary fibrosis is also characterized by an exacerbated type 2 immune response [58], as well as irreversible destruction and remodeling of the lung architecture as a consequence of the excessive deposition of collagen and other components of the ECM [59,60].

In the lung region, there are two large populations of macrophages. On the one hand, the alveolar macrophages are considered as resident macrophages of the lung tissue and are in contact with the alveoli epithelial cells, both type I and II. In fact, these macrophages are the first line of innate immune defense in the lungs and remain viable for a long time to preserve tissue homeostasis. On the other hand, interstitial macrophages have a shorter half-life and are derived from bone marrow monocytes. In addition, they are in the parenchyma between the microvascular endothelium and the epithelium [61,62].

The origin of the phenotypic subpopulations of macrophages is not yet well-understood. Macrophages may come directly from the maturation of monocytes, or from the transition based on phenotypically specialized macrophages due to their high plasticity and ability to adapt to the microenvironment. Of interest, depletion of inflammatory M1 macrophages has been shown to attenuate pulmonary fibrosis. This fact reinforces the idea that pro-fibrotic M2 macrophages are mainly derived from pro-inflammatory M1 macrophages. However, it is important to consider that they also come from monocytes CX3CR1+ and low levels of lymphocyte antigen 6 complex, locus C1 (Ly6C) in mice, which reach the lung via blood [59].

In physiological situations, alveolar macrophages produce low levels of inflammatory cytokines, maintaining their phagocytic activity and suppressing inflammation and adaptive immunity [61]. However, current evidence suggests that in patients with pulmonary fibrosis, there is an imbalance in the activity of subpopulations of macrophage phenotypes (M1 and M2), which plays a key role in the pathogenic response. In fact, the overactivation of macrophages, specifically the persistent increase in M2 macrophages, leads to the excessive release of pro-fibrotic mediators such as TNF-α, IL-1, IL-4, IL-10, IL-13, IL-17, IL-33, CXCL9, fibroblast growth factor (FGF), fibronectin, fractalkine, and CCL18, as well as growth factors, such as connective tissue growth factor (CTGF), TGF-β, TGF-α, platelet derived growth factor (PDGF) α, and M-CSF in the vicinity of collagen-producing fibroblasts. The secretion of all these mediators induces their proliferation and collagen production during the aberrant healing phase of fibrogenesis [63]. In addition, non-functional pro-inflammatory M1 macrophages, which positively regulate inflammation through the release of cytokines and inflammatory mediators [59], are incapable of producing the antifibrotic cytokine CXCL10 or matrix metalloproteinase (MMP), substances that degrade fibrotic tissue deposition. This imbalance, together with the recruitment of immune cells in the lung parenchyma and alveoli [61], causes persistent lung inflammation.

The unbalanced M1–M2 ratio is responsible for inducing and exacerbating lesions and fibrosis, remodeling tissue, and deregulating wound repair. This highlights the close regulatory relationship in both phenotypes. Definitely, it produces a worsening of the typical pathogenesis in patients with pulmonary fibrosis [59]. For this reason, current pulmonary fibrosis research is focused on cell surface markers and transcriptional profiles to identify the key role of different macrophage populations and their activation states in lung injury and repair [61].

In this way, the transcription factor Fos-related antigen-2 (Fra-2), encoded by Fosl2, has been highlighted. Fra-2 is co-localized in alveolar macrophages (described in Table 1), and it is involved in the transcription of type VI collagen (ColVI). This transcription factor has been reported to be upregulated and even correlated with ColVI and genes related to M2 activation, such as CD206, in IPF lung sections [58,64,65]. In vitro studies have proved that macrophages can promote myofibroblast activation in a ColVI and Fra-2 dependent manner [58,66]. This fact is corroborated by studies in murine models where it has been observed that genetic modification for the ectopic overexpression of Fra-2 leads to the development of spontaneous systemic fibrosis, predominantly affecting the lungs (Table 1). Besides, the inactivation of Fra-2, either by knockout (KO) murine models or by the administration of Fra-2/AP1 inhibitors, has been shown to protect them against bleomycin-induced pulmonary fibrosis. Nevertheless, it is important to take into account that neither macrophage recruitment nor alternative polarization was affected [58]. So, despite having a key pro-fibrotic role, Fra-2 is not essential for the polarization of M2 macrophage, but its expression seems to be important for their fibrotic activity [58,65].

The sphingosine-1-phosphate receptor-2 (S1PR2) is being extensively studied due to its association with pulmonary fibrosis and its pro-fibrotic role. It is expressed in alveolar macrophages, vascular endothelial cells, and alveolar epithelial cells. For instance, in a murine model of belomycin-induced pulmonary fibrosis, authors observed the accumulation of macrophages in the bronchoalveolar lavage fluid. Most of them were Mac-3+ macrophages that overexpressed the S1PR2 receptor along with the gene expression of IL-13, downstream of phosphorylated STAT6, IL-4, and M2 macrophage markers such as arginase 1 (Arg1), Fizz1, CCL17, and CCL24 (Table 1). In contrast, the blocking or gene suppression of the S1PR2 receptor resulted in an attenuation of fibrosis through a decrease in both STAT6 phosphorylation and the expression of M2 markers. Therefore, this evidence indicates that the presence of the S1PR2 receptor in macrophages worsens pulmonary fibrosis, a priori, through the STAT6 signaling pathway, and that it is related with macrophage polarization towards M2 [67].

The study conducted by Gharib et al. reported the involvement of the MMP epilysin (MMP-28) in both macrophage recruitment and polarization [68]. Firstly, they used macrophage polarized towards M1 or M2 from wild type (WT) and KO mice for MMP-28. They observed that MMP-28 was able to restrict the recruitment of neutrophils and macrophages biased towards a reparative phenotype. Furthermore, the absence of MMP-28 in macrophages polarized towards M1 (stimulated with LPS) in a KO murine model induced a significant increase in the expression of pro-inflammatory genes (Table 1), accentuating the pro-inflammatory state of macrophages. In contrast, its absence in macrophages polarized towards M2 (stimulated with IL-4/IL-13) produced a decrease in the polarized response towards M2, reflected in the expression of the markers Arg1, IL-10, and Fizz1, together with a decrease in the expression of TGF-β1. These data indicate that MMP-28 contributes to the pro-fibrotic response in the lung by promoting MMP-28-dependent M2 polarization and reducing the recruitment of repair cells. In line with these findings, in a KO murine model for MMP-28 of bleomycin-induced pulmonary fibrosis, they observed that the absence of MMP-28 was related to greater recovery of body weight, greater survival, and a reduction in polarization towards M2 characterized, in part, by a reduction in TGF-β1 expression and collagen synthesis. Ultimately, the data show a moderate level of protection against bleomycin-induced lung fibrosis. These results suggest that the regulation of macrophage function by MMP-28 has important implications in lung biology and wound repair mechanisms [68].

Recently, in the study by Wang et al., it was observed that methyl-CpG-binding domain protein 2 (MBD2) was altered in macrophages both in patients with IPF and in mice with bleomycin-induced pulmonary fibrosis [69]. Scientific evidence in murine models suggests that MBD2 represses SHIP phosphatase expression, enhancing PI3K/Akt signaling, therefore promoting polarization towards M2 macrophage [69,70]. On the one hand, MBD2 depletion protected against bleomycin-induced pulmonary fibrosis by a significant attenuation of TGF-β1 production, as well as a significant reduction in the accumulation of CD206+CD68+F4/80+ macrophage (Table 1), but not of M1, in the lung. On the other hand, the intratracheal administration of liposomes loaded with MBD2 small interfering RNA (siRNA) protected mice from lung lesions and bleomycin-induced fibrosis [69].

All of these studies suggest that a predominant pro-fibrotic M2 profile is detrimental to pulmonary fibrosis, led mainly through the release of TGF-β.

## 4. Macrophages in Heart Fibrosis

Cardiac fibrosis is a pathological disorder considered a common component of most cardiovascular diseases. It includes different types of fibrosis, such as replacement fibrosis, interstitial fibrosis, and perivascular fibrosis [71,72]. Cardiac fibrosis is characterized by an imbalance between the production and degradation of ECM in the myocardium. As a result, accumulation of scar tissue, distorted cardiac architecture, and cardiac dysfunction, which prevents adequate contraction and relaxation of the heart, occurs [73]. Due to the poor regenerative capacity of the myocardium, it loses much of its functionality when an episode that involves cardiomyocyte death occurs and those cells are replaced by a collagen scar [71].

Nowadays, cardiovascular diseases are the leading cause of death worldwide and cardiac fibrosis is implicated in almost all forms of these diseases [74]. However, there are still no effective therapies to inhibit or reverse cardiac fibrosis, mainly due to the complexity of the cell types and signaling pathways involved [72].

Although it is widely described that activated myofibroblasts are the main effector cells in the fibrotic heart, there are other cell types, such as macrophages, that can also contribute to the fibrotic response. They exert a crucial activity, as has been demonstrated in animal models [75,76,77], either by secreting key fibrogenic mediators, or through the differentiation of macrophages and subsets of monocytes into fibroblasts that infiltrate the injured heart among many other actions [71,78]. In line with the relevance of macrophages in cardiac fibrosis, several studies observed the accumulation of resident macrophages in damaged areas after experiencing a stressful situation for the heart. Furthermore, these macrophages come mainly from the recruitment of blood monocytes, although they can also come from the proliferation of local macrophages [79,80].

At steady state, the heart possesses a discrete subset of resident macrophages [79]. During the early inflammatory phase of myocardial infarction healing, monocytes with pro-inflammatory, phagocytic, and proteolytic properties are recruited. In contrast, during the reparative phase, monocytes with anti-inflammatory and angiogenic activity are recruited. This can result in the generation of multiple macrophage populations with distinct properties that mediate pro-inflammatory, anti-inflammatory, or fibrogenic actions due to the complexity of environmental conditions after cardiac injury. The relative contribution of monocytes and macrophages (and their respective subpopulations or phenotypes) in the cardiac fibrotic response depends on the pathophysiological basis of cardiac fibrosis [71].

Therefore, it is a priority to understand the cellular biology of the fibrotic response through the characterization of the macrophage subpopulations, their respective functions, and the identification of new factors involved in the modulation of pro-inflammatory/reparative responses in cardiac fibrosis. All of this will allow us to find new therapeutic strategies to face cardiac fibrosis.

Current evidence suggests that M2 macrophages improve cardiac fibrosis by modulating a large number of inflammatory mediators such as IL-10, Fizz1, Ym1, TGF-β, Arg1, IL-1β, TNFα, and NF-κB (Table 2). Experimentally, the studies in murine models by Shintani et al. and Jung et al. showed that prolonged treatment with IL-4 or IL-10, potent inducers of alternative macrophage activation, managed to significantly increase the amount of CD206+F4/80+ and Ly-6G-CD11b+ macrophage, respectively (Table 2). Specifically, IL-10 can exert inhibition of Hyal, interrupting the hyaluronic acid degradation, with an unbalanced ratio of collagen I and III. These changes result in improved cardiac repair, function, and remodeling [81,82]. However, other studies using neutralizing antibodies to IL-4 [83], or some components of the ECM previously mentioned, such as short oligosaccharides derived from hyaluronic acid [84] or recombinant type I and III collagen [85], observed an attenuation of cardiac fibrosis independently of TGF-β and an improvement in the disease, respectively. Therefore, it seems that both IL-4 and collagen could have a dual fibrotic role, possibly through the polarization of macrophage [81,82,83,84,85].

In line with those studies, Rickard et al. and Usher et al. showed that the mineralocorticoid receptor (MR), considered an important checkpoint in macrophage polarization, allows their differentiation towards the M1 phenotype and exacerbates cardiac fibrosis. In fact, in L-NAME/Angiotensin II (Ang-II) murine models lacking the receptor, and in vitro studies that used MR antagonists, an alternative M2 activation profile was observed (Table 2), showing protection against cardiac hypertrophy, fibrosis, and vascular damage. Of interest, they also observed that PPARγ agonists have the same effect as MR antagonists or KO models. Taking all together, the polarization towards F4/80+CD68+ M2 macrophage due to the inactivity of MR decreases cardiovascular inflammation and fibrosis [86,87].

On the other hand, the class A scavenger receptor has aroused interest due to its capacity to modulate macrophage polarization toward the M2 profile and its beneficial influence in cardiomyocyte necrosis after myocardial infarction. Therefore, this receptor has a close association with the inflammatory process of diseases that can cause myocardial infarction. The study by Hu et al. shows that this receptor has a cardioprotective role by suppressing the infiltration and polarization of the CD11c+ M1 macrophage dependent on this receptor (Table 2). Conversely, its inhibition or deletion reduces the polarization of CD11c− M2 macrophages and manages to impair cardiac function and exacerbates cardiac fibrosis [88].

Recently, in the work of Li et al., elevated levels of the macrophage marker CD226, which is strongly implicated in M2 polarization, have been observed in post-infarcted cardiac tissue. In fact, the study shows that the deletion of CD226 in murine F4/80+CD68+ macrophages favors polarization towards repairing CD206+ M2, while it suppresses polarization towards Mac-3 M1 (described in Table 2). Therefore, the absence of the CD226 marker induces an improved healing microenvironment and might be an attractive pharmacological target [89].

All of these studies suggest that a restorative M2 profile is beneficial against cardiac fibrosis. However, it should be noted that excessive M2 activity has also been related to excessive fibroblast activation, high collagen production, and ultimately, exacerbation of various types of fibrosis [58,80]. Indeed, in the study by Yang et al., KO mice for serum-glucocorticoid regulated kinase 1 (SGK1) showed a significantly reduced F4/80+CD11b+CD45+ macrophage accumulation and exhibited attenuation of Ang-II-induced cardiac fibrosis relative to WT mice. They observed that SGK1 played a key role in cardiac fibrosis through the phosphorylation and nuclear localization of STAT3, the promotion of the differentiation of macrophages towards CD206+ M2, their infiltration to the affected area, and the expression of pro-fibrotic cytokines (Table 2). All of this contributed to the transition from fibroblast to myofibroblast and consequently, the production of collagen, cardiac remodeling, and the development of cardiac fibrosis [90].

At this point, it is necessary to emphasize that there are several sub-profiles of M2 macrophage with totally different functions, whose balance and coordination during the disease are key events for an adequate recovery. Perhaps, in studies where an M2 profile is beneficial for fibrosis [81,82,83,84,85,86,87,88,89], it is a consequence of both an enhancement of antifibrotic activity and a reduction of pro-fibrotic activity. In contrast, considering the previously cited study by Yang et al., it is possible that the elimination of SGK1 reduces the polarization and infiltration of fibrotic M2 macrophage, associating this profile with an increase in cardiac fibrosis [90]. For this reason, it is vital to understand how the macrophage polarization towards a specific M2 sub-profile, which exerts a restorative activity avoiding fibrotic tissue accumulation, occurs. However, it is complex to establish limits between the different macrophage subtypes due to the high plasticity capacity of macrophage.

Following the same point of view, M1 macrophages are considered mainly a detrimental element for the development of fibrosis, but they also secrete MMPs that promote collagen degradation, a factor that has been implicated in multiple diseases [91,92,93].

## 5. Macrophages in Liver Fibrosis

Liver fibrosis is the result of the continuous and progressive activation of different liver-resident and infiltrating immune cells, which occurs in response to acute or chronic cell injury, and which perpetuates inflammation [94,95]. Chronic liver injury may arise as a response to the aggression of toxics such as alcohol, infections, or fat accumulation, which characterize alcoholic liver disease, viral hepatitis, or non-alcoholic fatty liver diseases, among others [96]. As it occurs in other scenarios, fibrogenesis starts as a defensive wound-healing mechanism, but becomes a persistent and pathogenic dysregulated tissue repair that leads to fibrillar connective tissue and fibrotic scar deposition, which accumulate in the liver parenchyma, disrupting its architecture and function and impeding tissue regeneration. If this fibrotic process continues, cirrhosis development becomes imminent, which may also lead to hepatocellular carcinoma and even liver failure [97].

Although hepatic fibrogenesis is a complex mechanism that involves the participation of a wide range of cell types, macrophages play a key role in both the development and regression of liver fibrosis. As immune cells, they regulate hepatic homeostasis and participate in the first steps of the inflammatory response to liver damage. In addition, they show a dual role participating in both the evolution of liver fibrosis and scar tissue degradation, and subsequent fibrosis resolution [97]. In the liver, two main types of macrophages can be distinguished. One type is a group of resident macrophages called Kupffer cells (KCs), which are found in hepatic sinusoids [98]. As resident macrophages, they develop pro-inflammatory and immunoregulatory roles participating in the beginning of the inflammatory response and maintaining hepatic homeostasis [99,100]. The other type is a group of hepatic macrophages known as MoMFs, among which bone-marrow derived macrophages (BMDMs) are included and are the most widely described. They come from peripheral blood monocytes that arrive to the liver in order to supply macrophage populations when required. Of interest, there are two different subpopulations of MoMFs. In murine studies, they are classified according to the expression of Ly6C. Those that express Ly6C are recognized as Ly6C^high^ and are described as pro-inflammatory mediators, while those that do not express Ly6C are recognized as Ly6C^low^ and have been defined as restorers of tissue integrity [96,97].

As previously explained, liver macrophages can act as fibrosis mediators exacerbating the accumulation of scar tissue in different ways. In line with this, hepatic stellate cells (HSCs) are resident non-mesenchymal cells, which upon transdifferentiation become myofibroblast-like cells and are the main collagen producers in the liver. Indeed, the most described action driven by macrophages that contributes to liver fibrosis is the activation of HSCs [94,95,101]. Liver macrophages can receive a wide variety of stimuli, which induces the secretion of certain substances that in turn activate the pro-fibrotic role of HSCs in remodeling the immune microenvironment and promoting ECM deposition [97]. Experimentally, it has been demonstrated that the removal of macrophages by using different techniques, such as genetic (LysM, myeloid-specific) models or clodronate liposomes in murine models, reduces liver injury and inflammation [102,103]. In this section of the review, we will describe in detail which stimuli activate liver macrophages, which phenotypes they have, and which response activates HCSs perpetuating fibrosis. This information is summarized in Table 3.

First, TGF-β activates the pro-fibrotic activity of HSCs when secreted by liver macrophages with different phenotypes. Interestingly, when CD11b+ KCs are activated by activin-A, they secrete TGF-β and TNF-α, which in turn promote the migratory capacity and increase the expression of α-SMA in HSCs [104]. In addition, TGF-β-mediated HSC activation, together with another chemokine known as PDGF-β, by liver F4/80+ macrophages was also observed after in vitro stimulation with oncostatin M, as well as in a thioacetamide (TAA)-fed animal model [105]. Indeed, TGF-β-mediated activation of both primary and immortalized HSCs (LX-2) was also observed with F4/80+ BMDMs, which were present in the liver tissue of different animal models such as high fat and high cholesterol (HFHC) model or a methionine-choline deficient (MCD) diet used to study non-alcoholic steatohepatitis (NASH)-related fibrosing steatohepatitis. In these models, complement cascades were also responsible for HSC activation, which increased the expression of metallopeptidase inhibitor (TIMP) 1, TIMP2, TGF-β1, collagen deposition, and endoplasmic reticulum stress markers GPR78, IRE1α, and PDI [26]. Furthermore, the Ly6C^high^ phenotype of monocyte infiltrating hepatic macrophages handles the activation of HSCs through TGF-β together with IL-13 [101,106,107,108,109]. Finally, a current and elegant study has demonstrated that TGF-β is released through the ERK signaling pathway by liver macrophages classified as F4/80^high^CD11b^low^CLEC4F+ KCs, when they are activated with c-Mer tyrosine kinase (MERTK), inducing HSC fibrotic effects in NASH [110].

Next, other cytokines that drive the activation of HSCs are TNF-α and IL-1β. In the presence of damage-associated molecular patterns (DAMPs), F4/80+ hepatic macrophages secrete such cytokines, inducing, through NF-κB signaling pathways, the proliferation of HSCs [101]. In addition, murine Ly6C^low^/+ macrophages also secrete TNF-α and IL-1β to activate HSCs, as well as IL-6. These cytokines favor HSC proliferation and induce the synthesis of TIMP1, which inhibits MMPs [111]. Interestingly, in the carbon tetrachloride (CCl4)-induced fibrosis animal experimental model, it was observed that this cytokine secretion was stimulated by the upregulation of TNF-like ligand 1 A (TLA1A), both TNF-α and IL-1β, and also PDGF-BB in BMDMs, which were responsible for enhancing activation and proliferation of primary HSCs [112]. Indeed, an interesting study demonstrated that in this CCl4-fibrosis animal model, KCs through a DAMP known as high-mobility group box-1 (HMGB1) could increase the expression of collagen type I alpha1 chain (COL1A1) by HSCs via phosphorylation of mitogen-activated protein kinase (MAPK). Of interest, in this study, authors demonstrated that the removal of HMGB1 inhibited the stimulation of COL1A1 by HSCs [113].

Following the description of substances that stimulate macrophages to modulate fibrosis development, CCL5 is an interesting mediator secreted by KCs in the viral infection caused by hepatitis virus C (HVC). Under its presence, both primary and immortalized HSCs show increased synthesis of fibrotic markers [114]. In line with this, CCL2 is another chemokine secreted by KCs that has been recently demonstrated to induce CCR2+ inflammatory monocyte infiltration through CCR2 receptors, which are responsible for HSC induction [115]. In fact, CCR2+ monocytes have become a marker whose levels increase as fibrosis progresses [116].

The last substance to be described is the glycoprotein granulin, which is synthetized by hepatic infiltrating monocytes in liver metastasis due to pancreatic ductal adenocarcinoma. This compound has been demonstrated to drive the evolution of quiescent HSCs to myofibroblasts that express periostin, perpetuating the pro-fibrotic microenvironment [117].

Apart from all cytokines and chemokines that have been described up to now, it has been recently published that in a TAA-induced fibrosis model, the P2X7R-NLRP3 pathway mediated macrophages infiltration in the liver. Once these macrophages are in the liver, they are responsible for the activation of HSCs by secreting IL-1β [118].

It is also relevant to consider that while hepatic macrophages are responsible for activating HSCs to produce fibrotic debris, HSCs can also stimulate KCs to continue activating themselves, contributing to a positive feedback regulation [87]. In this way, HSCs secrete Wisteria floribunda agglutinin-positive Mac-2 binding protein (WFA+-M2BP), which activates the expression of Mac-2 (Galectin-3) in KCs, a substance which in turn induces and perpetuates the activation of HSCs [119,120].

Another way in which macrophages contribute to fibrosis development is by interacting with natural killer (NK) T cells. In this case, hepatic macrophages regulate the migration of NKT cells to liver injury sites through the interaction of chemokine receptor CXCR6, present in NKT cells, with ligand CXCL16, which is highly expressed in both resident and infiltrating hepatic macrophages. Their accumulation in the hepatic environment results in the secretion of pro-inflammatory and pro-fibrotic cytokines that perpetuate inflammation and fibrosis [121]. For instance, it has been demonstrated that in hepatitis B viral infection, NKT cells tend to accumulate in the site of hepatic injury and secrete IL-4 and IL-13, which in turn activate HSCs [122].

As we have seen in several examples before, apart from resident macrophages, other types of macrophages can be recruited to the site of injury in the liver, potentiating fibrosis as a result [97]. In a CCl4-fibrosis murine model, BMDMs were found to be recruited via cannabinoid receptor 1 (CB1), which has also been demonstrated to be involved in the expression of pro-fibrotic and pro-inflammatory cytokines [123]. In the same animal model and in a bile duct ligation (BDL)-induced fibrosis model, BMDM infiltration occurred via CCR1 [124]. Interestingly, in an experimental model of rats fed with TAA, splenic red pulp macrophages were identified as TGF-β1 producers, which favor fibrogenesis [125]. Moreover, in a CCl4-fibrosis model, splenic macrophages could influence CCL2 secretion via SOCS3 of hepatic macrophages to promote macrophage infiltration and the accumulation of scar tissue [126].

It is widely known that macrophages contribute to not only fibrosis development, but also its resolution. The disease state and the differentiation status of macrophages are crucial to determine whether macrophages are playing a pro-fibrotic role or, in contrast, exerting anti-fibrogenic activity [94]. In this case, as previously described, it has been extensively demonstrated in murine models that macrophages of the Ly6C^low^ phenotype contribute to collagen breakdown and regression of ECM deposition [96,109,127]. An example of macrophages that have this phenotype are CD11b^high^/F4/80^intermediate^LY6C^low^ macrophages [95]. These specific macrophages secrete MMPs such as MMP-9 and MMP-12, which inhibit scar tissue deposition as well as prevent inflammation by inducing HSC apoptosis [128]. Triggering receptor expressed on myeloid cells (TREM)2+CD9+ scar-associated macrophages (SAMs) are another phenotype that secretes MMPs contributing to fibrosis resolution and inflammation restoration, in this case MMP-13, mediated by macrophage migration inhibitory factor (MIF) which is characteristic of early stages of disease [129,130]. Of interest, in both CCl4- and BDL-induced fibrosis experimental models, it has been shown that VEGF secreted by SAMs is able to increase the expression of other MMPs such as MMP-2 and MMP-14, as well as inhibit TIMP-1 and TIMP-2 [131]. In addition, recent studies have demonstrated, through murine models of liver damage and in human samples, the possibility of transforming a monocyte phenotype to an anti-inflammatory type through a non-canonical form of autophagy described as LC3-associated phagocytosis. Through this process, pro-inflammatory and pro-fibrotic pathways in the liver are inhibited [132].

Interestingly, certain murine model studies have revealed the possibility of switching from the pro-fibrotic Ly6C^high^ phenotype to the pro-restorative Ly6C^low^ through different molecular pathways [96]. One of the most described pathways involves the STAT-3-IL-10-IL-6 axis [133]. In contrast to that previously explained, IL-4 and IL-13 can also activate PtdSer-dependent receptor tyrosine kinases (RTKs) AXL and the proto-oncogene MERTK to lead the transition of macrophages into an anti-inflammatory and anti-fibrotic phenotype [134]. Moreover, in the CCl4-fibrosis model, this switch was induced by the soluble glycoprotein CD5L, which results in anti-fibrotic activity [135]. Of interest, it has also been demonstrated in both CCl4- and MCD diet induced fibrosis murine models that when macrophages express the scavenger receptor stabilin-1, the transformation of macrophages into Ly6C^low^ is favored [136].

To sum up, liver fibrosis is a complex mechanism that involves a wide variety of cell types and mediators. The presence of activated HSCs characterizes this type of fibrosis since they are not only common myofibroblasts, but have self-identity. Further studies are still needed to better characterize their activity. Moreover, as liver fibrosis occurs in other scenarios, there is still controversy over the exact macrophage phenotype that can be modulated to improve fibrogenesis. Thus, it would be interesting to have additional studies that consider the use of human samples to extend previous findings.

## 6. Macrophages in Kidney Fibrosis

Kidney fibrosis, as previously described for other scenarios, is an excessive pathological response characterized by increased wound healing and consequently, a great deposition and accumulation of ECM [137]. Such an increase in fibrotic tissue is frequently localized, in the space between tubules and peritubular capillaries, known as renal interstitial fibrosis [138], or in the glomerulus, leading to glomerulosclerosis [139]. As a result, atrophy of renal tissue, as well as narrowing of capillaries predominantly emerges. Indeed, this pathological response commonly appears in many chronic kidney diseases including diabetic nephropathy, hypertensive nephropathy, primary chronic glomerulonephritis, chronic interstitial glomerulonephritis, and chronic tubular disease, and may be the cause of renal failure and death [138,139]. Therefore, renal fibrosis arises as an attempt to repair the tissue in a context of chronic kidney inflammation, which may initiate as a response to different stimuli such as toxins, xenobiotics, infections, or genetic disorders, and become pathologically persistent [140], predisposing the patient to develop any form of the chronic kidney diseases previously mentioned [141].

In this context, the excessive activation of myofibroblasts, pivotal producers of ECM components and which highly express α-SMA, is the key event in the development of renal fibrosis [138]. In physiological conditions, fibroblasts contribute to maintaining interstitial matrix homeostasis. However, under pathological conditions, the presence of a huge range of substances mediates their activation into myofibroblasts, giving rise to fibrogenesis [142].

Again, among the wide variety of substances and stimuli that participate in myofibroblast activation, macrophages are commonly involved. Indeed, macrophages themselves can even transdifferentiate into active myofibroblasts in a process known as MMT [139]. In the renal environment, as immune cells, macrophages play a crucial role in the inflammatory response as well as tissue repair. First of all, both resident and infiltrating macrophages participate in any kind of renal injury, but the problem arises when the secretion of wound-healing factors, including TGF-β, persists in time, giving rise to pathological fibrogenesis. Two different phenotypes of macrophages can be distinguished in the renal environment. On the one hand, as previously explained, M1 macrophages (CD11b+/Ly6C^high^), which highly express iNOS, IL-12, IL-23, and Ly6C, are characteristic of early stages of the inflammatory process, by secreting TNF-α, IL-1β, IL-6, IL-15, and IL-18, among others. On the other hand, M2 macrophages (CD11b+/Ly6C^int^), which highly express Arg1, CD206, and chitinase-like proteins such as Ym1, Fizz1, and CD36, are in charge of repairing the affected tissue by secreting TGF-β, CCL17, CCL18, and CCL22, among others. The transition from one phenotype to the other is driven by the presence of high levels of IL-4, IL-10, and IL-13 [141]. The prolonged activity of M2 macrophages (CD11b+/Ly6C^low^) is responsible for the exacerbated fibrotic tissue deposition and accumulation by producing PDGF, IGF-1, and CCL17 [143].

In this section of the review, we will describe in-depth how renal macrophages are involved in fibrotic tissue generation and also in the fibrolytic process. First, BMDMs that participate in kidney injury, especially those with the M2 phenotype, can contribute to fibrogenesis through MMT, in which CD68, α-SMA, and in some cases, Src, are upregulated [138]. MMT occurs through the TGF-β/Smad3 signaling pathway, and it represents the main source of myofibroblasts [144,145]. After acquisition of the myofibroblast phenotype, they contribute to fibrogenesis with the secretion of fibronectin and collagen [146,147].

Next, as summarized in Table 4, renal macrophages, through many different mechanisms, secrete substances that promote fibrogenesis through myofibroblast activation or the recruitment and infiltration of circulating monocytes, as well as fibrosis resolution. In the first place, it is widely described that M2 macrophages secrete TGF-β1, FGF-2 [148], PDGF [149], or Galectin-3 [150] to promote proliferation, survival, and activation of myofibroblasts, and the consequent deposition of ECM [143]. Interestingly, using a unilateral urethral obstruction (UOO) model of fibrosis, Kitamoto et al. demonstrated how F4/80+ macrophages contributed to renal fibrosis through TGF-β and TNF-α mechanisms [151]. Additionally, it has been recently demonstrated that in a cisplatin model of renal injury performed in rats, CD163+ M2 macrophages highly express TGF-β1 [152]. In line with this, a study performed in diabetes type 2 patients with nephropathy showed that the presence of CD163+ macrophages was associated with patients with renal injury such as interstitial fibrosis [153]. In addition, an in vitro study in which human tubular renal cells were stimulated with the chemokine CCL18, which has been demonstrated to be produced by CD68+ macrophages [154], revealed that this chemokine upregulates fibronectin production in tubular epithelium, contributing to renal fibrosis development in the context of diabetic nephropathy [155].

Furthermore, myofibroblast activation from epithelial cells in a process known as EMT, from endothelial cells in a process known as EndoMT, or even from pericytes or mesangial cells can be promoted by M2 macrophages through the secretion of IL-1, MMP-9, TGF-β1, Ang-II, PDGF, IGF-1, or FGF-2 [143,156,157]. An elegant study described the role of MOMA2+ cells, a type of infiltrating macrophage, in fibrosis generation through the increased expression of TNF-α, IL-1β, TGF-β1, and fibronectin, as well as increased levels of renal oxidative stress. The infiltration of this type of macrophage was found to be mediated by Ang-II [158].

Among other forms of recruitment, macrophages can be recruited to the renal site through TGF-β1, a process mediated by CCL2, since TGF-β1 can regulate and increase the expression of this chemokine [143]. Interestingly, several studies have revealed that when the receptor of CCL2, CCR2, is knocked out, fibrogenesis and diabetic nephropathies are attenuated [159,160]. Furthermore, it has been demonstrated through a UUO model of renal injury that B cells also play an important role in recruiting macrophages bearing the phenotype CD11b+Ly6-G−/F4/80+ and, consequently, contributing to renal fibrosis development [161]. Of interest, the MyD88-mediated signaling pathway participates in the recruitment of M2 macrophages, specifically the IL-10+CD206+CD11b^high^ subtype, in a UUO model, contributing to collagen deposition [162]. Furthermore, it has been widely described that the Notch signaling pathway in macrophages is involved in renal fibrosis [139]. In line with this, Jiang et al. demonstrated that inhibiting the transcription factor recombination signal binding protein-Jκ (RBP-J), which is involved in the activation of the Notch pathway, decreased macrophage recruitment and further activation, thus improving renal fibrosis [163]. Finally, it is also relevant to highlight that the protein HMGB1 plays a crucial role in M1 macrophage polarization. Although M1 macrophages are the main characters in the inflammatory process, their prolonged activity also contributes to fibrogenesis, as demonstrated in a UUO model [164].

To sum up, as previously explained, renal macrophages have been found to also play a role in fibrosis resolution, although the exact phenotype that undergoes this process is not deeply understood. In the same way in which it occurs in other scenarios, MMP participate in this process, since they induce ECM degradation. Nevertheless, it is important to highlight that depending on the stage of renal disease, the role of MMP may vary. For instance, Nishida et al. demonstrated in a UUO mouse model the role of MMP-2 secreted by F4/80+ macrophages in the fibrolytic process [165]. However, as previously named, secreted MMP-9 can also exert a role in EMT [166,167]. In certain studies, it was revealed that in this renal fibrosis model, when MMP-9 was inhibited, tubular cell EMT was avoided and as a consequence, fibrosis development in the kidney was diminished due to osteopontin cleavage inhibition [168].

However, apart from MMP, other processes can mediate the fibrinolytic process. Of interest, the degradation of fibrotic tissue accumulation also may be in part mediated by mannose receptor 2 (Mrc-2), which is capable of internalizing collagen. To demonstrate that, it was observed that the absence of Mrc-2 in mice contributed to an increased collagen deposition [169]. In the same way, the absence of Ang-II type 1 receptor (Agtr1) also aggravates renal interstitial fibrosis, proposing a role of this receptor in renal fibrosis resolution [170].

To conclude, although there are several studies that have been useful for a better understanding of renal fibrogenesis, more recent studies that may include human samples are required to improve our knowledge of this field. In contrast to other scenarios, in this case, the M2 phenotype is the key responsible for fibrotic tissue accumulation; hence, it could be interesting to achieve a deeper characterization of its activity. Moreover, MMT is a fundamental process in the development of renal fibrosis. Thus, a better characterization of the mediators involved in this process would be extremely helpful in order to find possible pharmacological targets.

**Table 4:** Table summarizing how and which types of macrophages are involved in kidney fibrosis. Macrophage-myofibroblast transition (MMT), Transforming Growth Factor-beta (TGF-β), Tumor necrosis factor-alpha (TNF-α), Interleukin (IL), High-mobility group box-1 (HMGB1), Chemokine C-C motif ligand (CCL), Angiotensin II (Ang-II), Matrix metalloproteinase (MMPs), Epithelial-to mesenchymal transition (EMT), Mannose receptor 2 (Mrc-2), and Ang-II type 1 receptor (Agtr1).

Macrophage	Fibrosis Mediator	Effect	Reference
BMDMs M2 ↓ Myofibroblasts	Fibronectin and collagen	Transdifferentiation of macrophages to myofibroblasts (MMT)	[138,143,144,145,146,147]
F4/80+ macrophages	TGF-β, TNF-α	Fibrogenesis	[151]
CD163+	TGF-β1		[152]
CD68+	CCL18		[155]
MOMA2+ cells	Recruited by Ang-II. Produce TNF-α, IL-1β, TGF-β1, fibronectin and renal oxidative stress		[158]
Unspecified subtype	CCL2	Macrophage recruitment and further fibrogenesis	[159,160]
CD11b+Ly6-G−/F4/80+	B cells		[161]
IL-10+CD206+CD11b^high^	MyD88-mediated signaling		[162]
CD11b+F4/80+	Notch pathway		[163]
M1	HMGB1	Fibrogenesis	[164]
F4/80+	MMP-2	Fibrosis resolution	[165]
F4/80+	MMP-9, osteopontin	Tubular cell EMT and further fibrogenesis	[168]
F4/80+	Mrc-2	Fibrosis resolution	[169]
F4/80+	Agtr1		[170]

## 7. Macrophages in Intestinal Fibrosis

Intestinal fibrosis is a common and inevitable complication of both subtypes of inflammatory bowel disease (IBD)—ulcerative colitis (UC) and Crohn’s disease (CD)—due to the persistent and chronic inflammation present in both diseases. It is important to consider that intestinal fibrosis, like in other organs, plays an essential role in wound healing and tissue repair. Nevertheless, the chronic inflammation leads to progressive fibrosis activation, which triggers the excessive deposition of ECM, scarring of several tissues, damage to the organ, and impairment of its function [171]. This exacerbated accumulation of ECM differs between UC and CD patients because, while in UC patients it is limited to the submucosal and mucosal layers of the large intestine, in CD patients the ECM components can involve the whole intestinal wall of the gastrointestinal tract [172]. Intestinal fibrosis presupposes the development of several complications, including stricture formation, perforation, and fistula formation, which in turn require surgery due to the lack of efficient pharmacological drugs that prevent these complications [173].

It is important to highlight that intestinal fibrosis represents one of the most severe complications, along with the fistula, associated with IBD patients. Specifically, although most CD patients initially exhibit a purely inflammatory status without complications, approximately 10% of CD patients present a fibrotic phenotype at the moment of diagnosis, and around 20% of CD patients will develop fibrosis within 20 years after diagnosis. In the case of UC, a range from 2% to 11.2% of UC patients develop a fibrotic phenotype [174]. Thus far, there are no specific markers that predict whether patients will develop intestinal strictures, and given the scarce knowledge about the molecular mechanisms involved in intestinal fibrosis, further studies are needed in order to better understand this complication.

As we have with the rest of fibrotic scenarios, we will focus specifically on macrophages and their role in the development of intestinal fibrosis, summarized in Table 5. In IBD patients, besides an increase in the number of macrophages present in inflamed tissue compared with non-IBD patients, there is also an alteration in the macrophage phenotype. In fact, the M2 CD206+ macrophages are significantly increased in CD patients compared with non-IBD patients [175]. In addition, these M2 macrophages are also higher in chronic patients compared with newly diagnosed patients [176], which reflects that these macrophages not only are present in inflamed tissues, but also increase their presence in chronic inflammatory areas. The functional role of M2 macrophages in intestinal wound healing was reported by our group, where we showed that STAT6−/− mice exhibited delayed wound healing in acute 2,4,6-Trinitrobenzenesulfonic acid (TNBS)-colitis associated with a reduced number of M2a macrophages and administration of M2a macrophages accelerated the intestinal regeneration and ameliorated acute colitis. In that study, we also demonstrated that specifically M2a macrophages express higher levels of Wnt ligands [177]. In contrast with other studies, in this case, it was possible to determine the specific M2 macrophage subtype responsible for such effects. Hence, further efforts could be made in order to better characterize the exact subtype of M2 macrophages responsible for the wide variety of effects described. Moreover, we have also reported that the lack of STAT6 favors intestinal fibrosis development after chronic administration of TNBS, associated with a reduction in the number of CD206+ and an increase in the number of CD16+ macrophages. In that study, we also demonstrated that CD patients presented an increased number of CD16+ macrophages, which express higher levels of Wnt6, and the administration of IL-4 treated macrophages, M2a macrophages, reduces intestinal fibrosis, revealing for the first time an anti-fibrotic role of M2a macrophages, specifically in intestinal fibrosis [178].

Macrophages exert a pivotal role in fibrosis evolution since growing evidence shows that these cells are able to secrete the central protagonist of fibrosis, TGF-β [179,180,181]. In addition, a deeper analysis of the macrophage phenotype responsible for the TGF-β revealed that specifically M2 macrophages secrete higher levels of this pro-fibrotic cytokine. In fact, not only do M2 macrophages release TGF-β, but they also, through this cytokine, induce the activation of different cell lines. For instance, it has been reported that M2 macrophages increases the stemness and migration of glioma cells through the SMAD2/3 pathway [182]. In line with this, a current study has described that M2 macrophages (CD206+ macrophages) secrete TGF-β and induce the phosphorylation of Smad3 in α-SMA+ cells during socket healing [183]. Nevertheless, given the scarcity of studies to our knowledge that have analyzed intestinal fibrosis specifically, whether M2 macrophages or a different macrophage phenotype can activate intestinal fibroblasts via TGF-β, is still an unresolved question that needs to be addressed. Therefore, although it might be extrapolated from different tissues that M2 macrophages exert a crucial role in intestinal fibrosis through TGF-β, further studies are needed in order to confirm this hypothesis.

Besides TGF-β, some cytokines recently have been associated with intestinal fibrosis development. Indeed, IL-36, a cytokine that is increased in both CD and UC patients, has been identified as an important activator of intestinal fibroblasts [184]. Of interest, it has been reported recently that IL-36A, mainly expressed in macrophages, is increased specifically in the fibrotic areas of CD patients, correlates with the inflammatory degree, and is found in CD14+, CD64+, and CD163+ macrophages. In addition, in this elegant study, the authors also showed that these IL-36+ cells are located close to α-SMA+ cells and type VI collagen, which strongly suggests that these cells might regulate fibroblast activation in IBD patients [185].

Another important cytokine that needs to be considered is IL-34, which is the second ligand of colony-stimulating factor-1 receptor (CSF-1R). It can polarize macrophages towards a phenotype similar to the tumor associated macrophages (TAMs), and it exerts a pleiotropic role in the regulation of immune and inflammatory processes since it stimulates the secretion of membrane-associated IL-1α, favors the switch of memory T cells into helper T cells (Th), expands the CD4+/CD8+ Foxp3+Tregs, and suppresses the function of both T and NK cells [186]. Of interest, it has been recently reported that this cytokine is enhanced in CD patients and that such increase is exacerbated specifically in those CD patients with a fibrotic behavior. In addition, in the same study, the authors demonstrated that IL-34 induces collagen expression in primary intestinal fibroblasts through the p38 kinase pathway, which strongly points to a crucial role for this cytokine in intestinal fibrosis [187].

**Table 5:** Table summarizing how and which types of macrophages are involved in intestinal fibrosis. 2,4,6-Trinitrobenzenesulfonic acid (TNBS), Interleukin (IL).

Macrophage	Fibrosis Mediator	Effect	Reference
M2a	Wnt ligands	Accelerates wound healing in acute colitis.	[177]
M2a	Wnt ligands	Reduces intestinal fibrosis induced by chronic TNBS administration.	[178]
CD16+	Wnt6	Favors intestinal fibrosis development.	[178]
CD14+, CD64+ and CD163+	IL-36	IL-36A+ macrophages might regulate the activation of intestinal fibroblasts.	[185]
M2	IL-34	Induces the expression of collagen in intestinal fibroblasts.	[187]

Taking these findings together, we can confirm that macrophages play a crucial role in intestinal fibrosis, and all the evidence seems to point to M2 macrophages as responsible for this complication.

## 8. Conclusions

Fibrosis is a pathophysiological process and a common complication in a wide variety of diseases that affect many different organs. Although the locations differ, this process shares a similar pattern in all areas affected, which involves the accumulation of connective tissue and, as a result, a loss in parenchymal integrity and dysfunction of the organ affected. Hence, as a complication that affects patients worldwide, fibrosis increasingly needs to be addressed, since at present, there are still no effective therapies to efficiently inhibit or reverse it.

Among the complex mechanisms that are involved in fibrogenesis, macrophages have been described as crucial candidates that modulate the fibrotic process. Although the main role of macrophages, as immune cells, is to maintain tissue homeostasis, as we have described within this review, their participation in fibrosis development is also essential. Through a wide variety of signaling pathways, they are responsible for the accumulation of connective tissue. As summarized during this review, several studies have demonstrated that removing specific types of macrophages results in an improvement in fibrosis. Controversially, they not only promote ECM deposition, but also handle fibrosis resolution.

Therefore, characterizing the exact macrophage phenotype that undergoes profibrotic or fibrolytic effects would be remarkable. At this point, different macrophage phenotypes have been identified that, depending on their type, microenvironment, and stimulus, are key participants and can act in one way or another. In certain cases, it is widely known which phenotype aggravates fibrosis. Nevertheless, in most of the cases, there is huge controversy over whether a particular phenotype aggravates or improves fibrosis. Thus, in order to better understand and differentiate among macrophage phenotypes and their activity, a deeper characterization is urgently needed to develop potential pharmacological targets against fibrosis.

Hence, it is a priority to understand the cellular biology of the fibrotic response through the characterization of macrophage subpopulations and their respective functions. It would be extremely interesting to identify all the macrophage-secreted cytokines involved in the activation or resolution of the fibrotic targets, because these molecules might be potential pharmacological targets against fibrosis.

Interestingly, improve techniques to characterize, identify, and isolate macrophages have allowed the identification of new markers and factors involved in the modulation of pro-inflammatory/reparative responses in fibrosis. Such identification makes possible the application of new therapeutic strategies to treat a common complication in several pulmonary pathologies, cardiovascular diseases, alcoholic or non-alcoholic fatty liver diseases, viral hepatitis, a wide variety of chronic kidney diseases, and IBD, as previously named. Even so, further efforts must be made to better understand the pathogenesis of fibrosis.

Taking these observations together, it is clear that additional studies of all types of fibrosis need to be performed in order to better elucidate the specific molecules coming from macrophages involved in fibrosis. Although we can confirm that macrophage phenotype is detrimental to their activity, there is still controversy over the exact phenotype that improves this complication. Thus, to achieve this ambitious goal, a better identification of macrophages and their activity is required to characterize them and develop an effective treatment.

## Figures and Tables

**Table 1:** Table summarizing how and which types of macrophages are involved in pulmonary fibrosis. Cluster of differentiation (CD), Fos-related antigen-2 (Fra-2), Transforming Growth Factor-beta (TGF-β), Matrix metalloproteinase (MMP), Chemokine C-C motif ligand (CCL), Mannose receptor 1 (CD206), Arginase 1 (Arg1), Collagen (Col), Macrophage anti-inflammatory/pro-fibrotic (M2), Sphingosine-1-phosphate receptor-2 (S1PR2), Signal transducer and activator of transcription (STAT), Interleukin (IL), Interferon regulatory factor (IRF), Connective tissue growth factor (CTGF), Resistin-like protein α (Fizz1), Class II major histocompatibility complex (MHCII), Chemokine C-X-C motif ligand (CXCL), Tumor necrosis factor-alpha (TNF-α), Macrophages pro-inflammatory (M1), Methyl-CpG-binding domain 2 (MBD2), and Small interfering RNA (siRNA).

Macrophage	Macrophage Polarizing Marker	Fibrosis Mediator	Effect	Reference
F4/80+ CD206+ CD11b^low^	Fra-2	TGF-β1, MMP12, CCL17, CCL22, CD206, Arg1, Ym1, and Ym2.	(1) Increases the activation of myofibroblast in a ColVI and Fra-2 dependent manner in vitro. (2) Specifically controls the fibrotic activity of M2 and leads to the development of spontaneous systemic fibrosis in murine transgenic Fra-2 model.	[58]
Mac-3+	S1PR2	STAT6, IL-13, IL-4, IRF4, CTGF, TGF-β1, Fizz1, Arg1, CCL17, CCL24, and Alox15.	Promotes fibrosis by increasing M2 markers and STAT6-dependent IL-13 and IL-4 expression in cells of bronchoalveolar lavage fluid (most macrophage).	[67]
F4/80+ MHCII+ CD11b^int^ CD45^int^ or F4/80+ MHCII+ CD11b^low^ CD45^low^	MMP-28	IL-6, CXCL1, CXCL2, TNF-α, IL-1β, IL-10, Col1a1, Fizz1, Arg1 and IL-10.	(1) Attenuates the pro-inflammatory state of M1 macrophage. (2) Promotes M2 polarization and reduces repair cell recruitment, TGF-β1 expression, and collagen synthesis. (3) Its absence offers moderate protection against bleomycin-induced pulmonary fibrosis in murine model.	[68]
F4/80+	MBD2	Not described.	Its absence reduces hydroxyproline levels and the fibrosis score, offering protection against fibrosis in several murine models.	[69]
F4/80+	MBD2	TGF-β1.	Its absence reduces TGF-β1 produced by Smad2/3 signaling pathway.	[69]
F4/80+ CD68+ CD206+	MBD2	Arg1, Fizz1, Ym1, IL-6.	Promotes specialization towards M2 macrophage in murine model.	[69]
F4/80+ CD68+ CD206+	MBD2	PI3K/Akt, SHIP.	It improves PI3K/Akt pathway by suppressing SHIP. This promotes specialization towards M2 macrophage.	[69]
F4/80+ CD68+ CD206+	MBD2	Arg1.	Level of fibrosis (hydroxyproline), expression of fibrotic markers (collagen, α-SMA), and M2 marker (Arg1) were reduced by the treatment with siRNA of MBD2.	[69]

**Table 2:** Table summarizing how and which types of macrophages are involved in cardiac fibrosis. Lymphocyte antigen 6 complex, locus C1 (Ly6C), Cluster of differentiation (CD), Transforming Growth Factor-beta (TGF-β), Interleukin (IL), Tumor necrosis factor-alpha (TNF-α), Arginase 1 (Arg1), Mannose receptor 1 (CD206), Mannose receptor 2 (Mrc-2), Chemokine C-C motif ligand (CCL), Macrophage anti-inflammatory/pro-fibrotic (M2), Hypoxia induced factor 1 alpha (HIFα), Vascular endothelial growth factor (VEGF), Chemokine C-X-C motif ligand (CXCL), Resistin-like protein α (Fizz1), Matrix metalloproteinase (MMP), Metallopeptidase inhibitor (TIMP), Coagulation Factor XIII A Chain (F13a1), Mineralocorticoid receptor (MR), Plasminogen activator inhibitor-1 (PAI-1), High temperature requirement A (Htra), Phosphoinositide-dependent kinase (Pdk), Cadherin-2 precursor (CDH2), Perosyxomel proliferator activated receptors (PPAR), Macrophages pro-inflammatory (M1), Apoptosis signal-regulating kinase 1 (ASK1), Nuclear factor-kappa B (NFkB), Serum/Glucocorticoid Regulated Kinase 1 (SGK1), Angiotensin II (Ang-II), and Signal transducer and activator of transcription (STAT).

Macrophage	Macrophage Polarizing Marker	Fibrosis Mediator	Effect	Reference
Ly-6G- CD11b+	Not described.	TGF-β1, IL-1β, Hyal3, TNF-α, Arg1, CD206 and CCL2.	Increases polarization of M2 macrophage leading to an overall anti-inflammatory response in the infarct region, improving cardiac fibrosis.	[81]
Ly-6G- CD11b+	Not described.	Hyal3.	It reduces hyaluronic acid degradation in the infarct.	[81]
CD206+ F4/80+	Not described.	IL-10, IL-1rn, HIF1α, VEGFα, CXCL12, Fizz1, Ym1 and TGF-β.	Increases polarization and amount of M2 macrophage, improving cardiac repair, function, and remodeling.	[82]
CD206+ F4/80+	Trib1.	Not described.	Its absence hinders improvement induced by IL-4 treatment through the depleted ability to develop M2 macrophage.	[82]
CD68+	Anti-IL-4	Not described.	Treatment with antibodies attenuated the increased macrophage numbers in fibrotic regions induced by IL-4.	[83]
F4/80+ CD206+	Not described.	VEGF, CCL2, and CXCL5.	Hyaluronic acid induces VEGF and chemokine release, and migration and polarization of macrophage toward M2.	[84]
F4/80+ Ly-6C^high^	Not described.	CD206, MMP1, MMP2, MMP9, TIMP1, TIMP2, and Arg1.	Recombinant type I and III collagen induce polarization of macrophage toward M2 and improve disease after myocardial infarction.	[85]
CD68+	Mineralocorticoid receptor	Not described.	Its absence protects against cardiac fibrosis, avoiding the increased number of infiltrating macrophage and the increased collagen induced by deoxycorticosterone in chronic murine model.	[86]
F4/80+	Mineralocorticoid receptor	F13a1, Arg1, Ym1, Fizz1 and TNF-α.	Blockade of the MR increases M2 marker expression and reduces the levels of TNF-α.	[87]
F4/80+	Mineralocorticoid receptor	TGF-β, PAI1, Htra1, Adm, Pdk4 and Cdh2.	Blockade of the MR increases antifibrotic and cardioprotective genes and decreases pro-fibrotic genes.	[87]
Unspecified subtype	PPARγ	TNF-α, Arg1, Ym1, and CCL17.	PPARγ promotes polarization of macrophage toward M2.	[87]
F4/80+ CD11c+ (M1) CD11c− (M2)	Class A scavenger receptor	ASK1, p38, NF-κB, IL-1β, IL-6, TNF-α, IL-10, Arg1 and Mrc-2.	Improves infiltration towards M2 and worsens polarization towards M1. This increases anti-inflammatory cytokine release, improving cardiac function deterioration and attenuating cardiac fibrosis in a murine model.	[88]
Ly-6C^high^ (proinfl) Ly-6C^low^ (antiinfl)	Class A scavenger receptor	Not described.	Improves infiltration of anti-inflammatory macrophage and worsens infiltration of pro-inflammatory macrophage.	[88]
F4/80+ CD68+ Mac-3	CD226	Mac-3, IL-1β, IL-6, and IL-12p40.	Deletion of CD226 reduces both M1 infiltration and markers.	[89]
F4/80+ CD68+ CD206+	CD226	CD206, Arg1, Fizz1, Ym1, and IL-10.	Deletion of CD226 both promotes M2 infiltration and markers and induces a restorative microenvironment.	[89]
F4/80+ CD11b+ CD45+	SGK1	Mac-2.	Its absence significantly reduced macrophage accumulation in hearts after Ang-II infusion.	[90]
F4/80+ CD206+	SGK1	CD206, TGF-β, IL-13, STAT3, TNF-α and IL-10.	Its absence reduces (i) M2 macrophage polarization and infiltration through STAT3, (ii) cardiac fibroblast transformation, and (iii) pro-fibrotic chemokine release, improving Ang-II-induced cardiac fibrosis.	[90]

**Table 3:** Table summarizing how and which types of macrophages are involved in liver fibrosis. Transforming Growth Factor-beta (TGF-β), Tumor necrosis factor-alpha (TNF-α), alpha-Smooth muscle actin (α-SMA), Hepatic stellate cells (HSCs), Platelet-derived growth factor (PDGF), Metallopeptidase inhibitor (TIMP), Bone marrow-derived macrophages (BMDMs), Interleukin (IL), Kupffer Cells (KCs), Lymphocyte antigen 6 complex, locus C1 (Ly6C), Nuclear factor-kappa B (NFkB), Extracellular signal-regulated kinase (ERK), High-mobility group box-1 (HMGB1), Wisteria floribunda agglutinin-positive Mac-2 binding protein (WFA+-M2BP) Collagen type I alpha1 chain (COL1A1), Chemokine C-C motif ligand (CCL), Chemokine C-C motif receptor (CCR), Chemokine C-X-C motif ligand (CXCL), Chemokine C-X-C motif receptor (CXCR), Natural Killer (NK), Cannabinoid receptor 1 (CB1), Matrix metalloproteinase (MMPs), Mitogen-activated protein kinase (MAPK), Scar-associated macrophages (SAMs), migration inhibitory factor (MIF), c-Mer tyrosine kinase (MERTK), Signal transducer and activator of transcription (STAT), and Receptor tyrosine kinases (RTK).

Macrophage	Fibrosis Mediator	Effect	Reference
CD11b+ KCs	TGF-β, TNF-α	Promotion of migratory capacity, and increased expression of α-SMA and collagen by HSCs.	[104]
F4/80+ hepatic macrophages	TGF-β, PDGF-β	Increased expression of TIMP1 in HSCs leading to myofibroblast differentiation.	[105]
F4/80+ BMDMs	TGF-β and complement cascades	Activation and proliferation promotion of primary HSCs and immortalized HSCs (LX-2 cells), which increased expression of TIMP1, TIMP2, TGF-β1, collagen deposition and endoplasmic reticulum stress markers GPR78, IRE1α and PDI.	[26]
Ly6C^high^	TGF-β, IL-13	Activation of HSCs to myofibroblasts.	[101,106,107,108,109]
F4/80^high^CD11b^low^CLEC4F+ resident macrophages (KCs)	TGF-β.	Activation of HSCs through ERK signaling pathway.	[110]
F4/80+ hepatic macrophages	TNF-α, IL-1β	Proliferation of HSCs induced through NFkB signaling pathways.	[101]
Ly6C^low^/+	TNF-α, IL-1β, IL-6	HSC activation, proliferation and synthesis of TIMP1.	[111]
BMDMs	TNF-α, IL-1β PDGF-BB	Enhanced activation and proliferation of primary HSCs.	[112]
KCs	HMGB1	Increased expression of COL1A1 by HSCs via MAPK phosphorylation.	[113]
KCs	CCL5	Increased synthesis of inflammatory (NLRP3, IL-1b, IL-6) and fibrotic markers (TGFb1, COL4A1, MMP2, α-SMA) by both primary and immortalized HSCs.	[114]
CCR2+ monocytes	CCL2	HSC activation induced by interaction with CCR2 receptor.	[115]
MAMs (CD45+CD11b+F4/80+)	Periostin	Evolution of quiescent HSCs to myofibroblasts, perpetuating the fibrotic microenvironment.	[117]
Murine experimental macrophages	IL-1β	HSC activation by macrophages infiltrated through P2X7R-NLRP3.	[118]
KCs	Galectin-3	HSCs secrete WFA+-M2BP, which activate KCs to secrete Galectin-3, which in turn induces and perpetuates the activation of HSCs.	[119,120]
Ly6C−F4/80++CD11b− KCs	CXCR6-CXCL16 interaction	Migration to liver injury sites by NK T cells that secrete pro-inflammatory and pro-fibrotic cytokines	[121]
BMDMs	CB1	Macrophage recruitment and fibrosis potentiation	[123]
	CCR1		[124]
Splenic red pulp macrophages	TGF-β1	Favor fibrogenesis.	[125]
CD68− splenic macrophages	CCL2	Promote macrophage infiltration and accumulation of scar tissue deposition.	[126]
CD11b^high^/F4/80^int^LY6C^low^	MMP-9 MMP-12	Inhibit scar tissue deposition and inflammation by inducing HSC apoptosis.	[128]
TREM2+CD9+	MMP-13	Fibrosis resolution and inflammation restoration through MIF	[129,130]
SAMs	MMP-2 and MMP-14 increase and TIMP-1 and TIMP-2 inhibition	Fibrosis resolution	[131]
Ly6C^high^ ↓ Ly6C^low^	STAT-3-IL-10-IL-6 axis	Switching from the pro-fibrotic Ly6C^high^ phenotype to the pro-restorative Ly6C^low^	[133]
	PtdSer-dependent RTKs and MERTK		[134]
	CD5L		[135]
	stabilin-1		[136]

## Data Availability

Not applicable.
